# Maintenance of Elective Patient Care at Berlin University Children's Hospital During the COVID-19 Pandemic

**DOI:** 10.3389/fped.2021.694963

**Published:** 2021-08-30

**Authors:** Nicolas Terliesner, Alexander Rosen, Angela M. Kaindl, Uwe Reuter, Kai Lippold, Marcus A. Mall, Horst von Bernuth, Alexander Gratopp

**Affiliations:** ^1^Charité—Universitätsmedizin Berlin, Corporate Member of Freie Universität Berlin, Humboldt-Universität zu Berlin, and Berlin Institute of Health, Department of Pediatric Respiratory Medicine, Immunology and Critical Care Medicine, Berlin, Germany; ^2^Charité—Universitätsmedizin Berlin, Corporate Member of Freie Universität Berlin, Humboldt-Universität zu Berlin, and Berlin Institute of Health, Department of Pediatric Neurology, Berlin, Germany; ^3^Charité—Universitätsmedizin Berlin, Corporate Member of Freie Universität Berlin, Humboldt-Universität zu Berlin, and Berlin Institute of Health, Center for Chronically Sick Children, Berlin, Germany; ^4^Charité—Universitätsmedizin Berlin, Corporate Member of Freie Universität Berlin, Humboldt-Universität zu Berlin, and Berlin Institute of Health, Institute of Cell Biology and Neurobiology, Berlin, Germany; ^5^Charité—Universitätsmedizin Berlin, Corporate Member of Freie Universität Berlin, Humboldt-Universität zu Berlin, and Berlin Institute of Health, Berlin, Germany; ^6^Berlin Institute of Health at Charité—Universitätsmedizin Berlin, Berlin, Germany; ^7^German Center for Lung Research (DZL), Associated Partner, Berlin, Germany; ^8^Department of Immunology, Labor Berlin GmbH, Berlin, Germany; ^9^Berlin Institute of Health Center for Regenerative Therapies, Berlin-Brandenburg Center for Regenerative Therapies (BCRT), Charité Universitätsmedizin Berlin, Berlin, Germany

**Keywords:** COVID-19, SARS-CoV-2, university children's hospital, elective patient care, decentralized admission, nosocomial infection, staff sick ratio

## Abstract

**Background:** In Germany, so far the COVID-19 pandemic evolved in two distinct waves, the first beginning in February and the second in July, 2020. The Berlin University Children's Hospital at Charité (BCH) had to ensure treatment for children not infected and infected with SARS-CoV-2. Prevention of nosocomial SARS-CoV-2 infection of patients and staff was a paramount goal. Pediatric hospitals worldwide discontinued elective treatments and established a centralized admission process.

**Methods:** The response of BCH to the pandemic adapted to emerging evidence. This resulted in centralized admission via one ward exclusively dedicated to children with unclear SARS-CoV-2 status and discontinuation of elective treatment during the first wave, but maintenance of elective care and decentralized admissions during the second wave. We report numbers of patients treated and of nosocomial SARS-CoV-2 infections during the two waves of the pandemic.

**Results:** During the first wave, weekly numbers of inpatient and outpatient cases declined by 37% (*p* < 0.001) and 29% (*p* = 0.003), respectively. During the second wave, however, inpatient case numbers were 7% higher (*p* = 0.06) and outpatient case numbers only 6% lower (*p* = 0.25), compared to the previous year. Only a minority of inpatients were tested positive for SARS-CoV-2 by RT-PCR (0.47% during the first, 0.63% during the second wave). No nosocomial infection of pediatric patients by SARS-CoV-2 occurred.

**Conclusion:** In contrast to centralized admission via a ward exclusively dedicated to children with unclear SARS-CoV-2 status and discontinuation of elective treatments, maintenance of elective care and decentralized admission allowed the almost normal use of hospital resources, yet without increased risk of nosocomial infections with SARS-CoV-2. By this approach unwanted sequelae of withheld specialized pediatric non-emergency treatment to child and adolescent health may be avoided.

## Introduction

In 2020 the COVID-19 pandemic has imposed unprecedented challenges on the global health care system. In Germany, the pandemic evolved as two distinct waves of SARS-CoV-2 infection with the first wave starting in February, 2020 and the second in July, 2020. In February, 2020, information on the clinical manifestation of SARS-CoV-2 infection in children was highly limited (1–3). Due to stricter quarantine measures in China compared to Germany it was difficult to predict how many children with SARS-CoV-2 infection were to be admitted to hospitals in Germany (4). At the same time, German governmental directives at country and federal state levels issued in March, 2020 requested hospitals to focus on fighting the pandemic and to interrupt elective patient care (5). There were only few reports from Italy, the second most affected country at that time, which strongly suggested triage and isolation measures, but also that most children infected with SARS-CoV-2 do not require intermediate or even intensive care (6–8).

The Berlin University Children's Hospital at Charité (BCH) is the largest children's university hospital in Germany that comprises all pediatric sub-specialties. It has a specialized pediatric care mandate for the Berlin-Brandenburg metropolitan region with a population of ~6.1 million including ~1.0 million children and adolescents. Devoid of the Departments of Neonatology and Child Psychiatry that imposed independent measures during the COVID-19 pandemic, BCH comprises 195 inpatient beds. In 2019, 10,483 inpatients and 65,501 outpatient cases with 126.642 outpatient presentations (34,563 of which were presentations to the Pediatric Emergency Department) were treated at BCH. BCH comprises three Pediatric Intermediate Care (IMC) wards, one Stem Cell Transplantation Unit, and one Pediatric Intensive Care Unit (PICU), specialized day clinics and outpatient clinics.

With its mandate for specialized pediatric care, BCH established the following aims in response of the COVID-19 pandemic. The first aim was to treat children and adolescents with COVID-19 that required hospital admission. The second aim was to maintain care for critically ill pediatric patients in need for tertiary or quaternary care. This included the prevention of nosocomial SARS-CoV-2 infections. The third aim was to prevent SARS-CoV-2 cross-infection of the hospital staff. During the first wave of the pandemic, we established centralized admission via a single ward exclusively dedicated to children and adolescents with unclear SARS-CoV-2 status to avoid nosocomial infections. In order to release staff (in particular nurses) and resources for the treatment of COVID-19 patients, we interrupted in- and outpatient admissions of any elective patients. During the first wave it became evident that SARS-CoV-2 infections in pediatric patients usually take a mild clinical course that does not require admission to hospital, so that most of the hospital's resources liberated at the beginning of the first wave remained unused (3, 4). Further, we observed no nosocomial infections with SARS-CoV-2 during the first wave. Based on this experience, BCH maintained elective, non-emergency tertiary and quaternary inpatient and outpatient care during the second wave of the pandemic. We also maintained a decentralized admission process to our wards.

The aim of this study was to determine and compare the effects of our adaptive measures to the COVID-19 pandemic during its first wave (i.e. only emergency treatment, no elective care; centralized admission) and second wave (i.e. continuing elective care; decentralized admission). We therefore compared the number of inpatients and outpatients treated during the two waves of the 2020 pandemic with the numbers of patients treated in 2019. Further, we compared year by year availability of nursing and medical staff. Finally, we determined the number of SARS-CoV-2 positive pediatric patients and of nosocomial infections of patients and staff.

## Methods

### Definition of First and Second Wave of COVID-19 Pandemic

**Figure 1:**
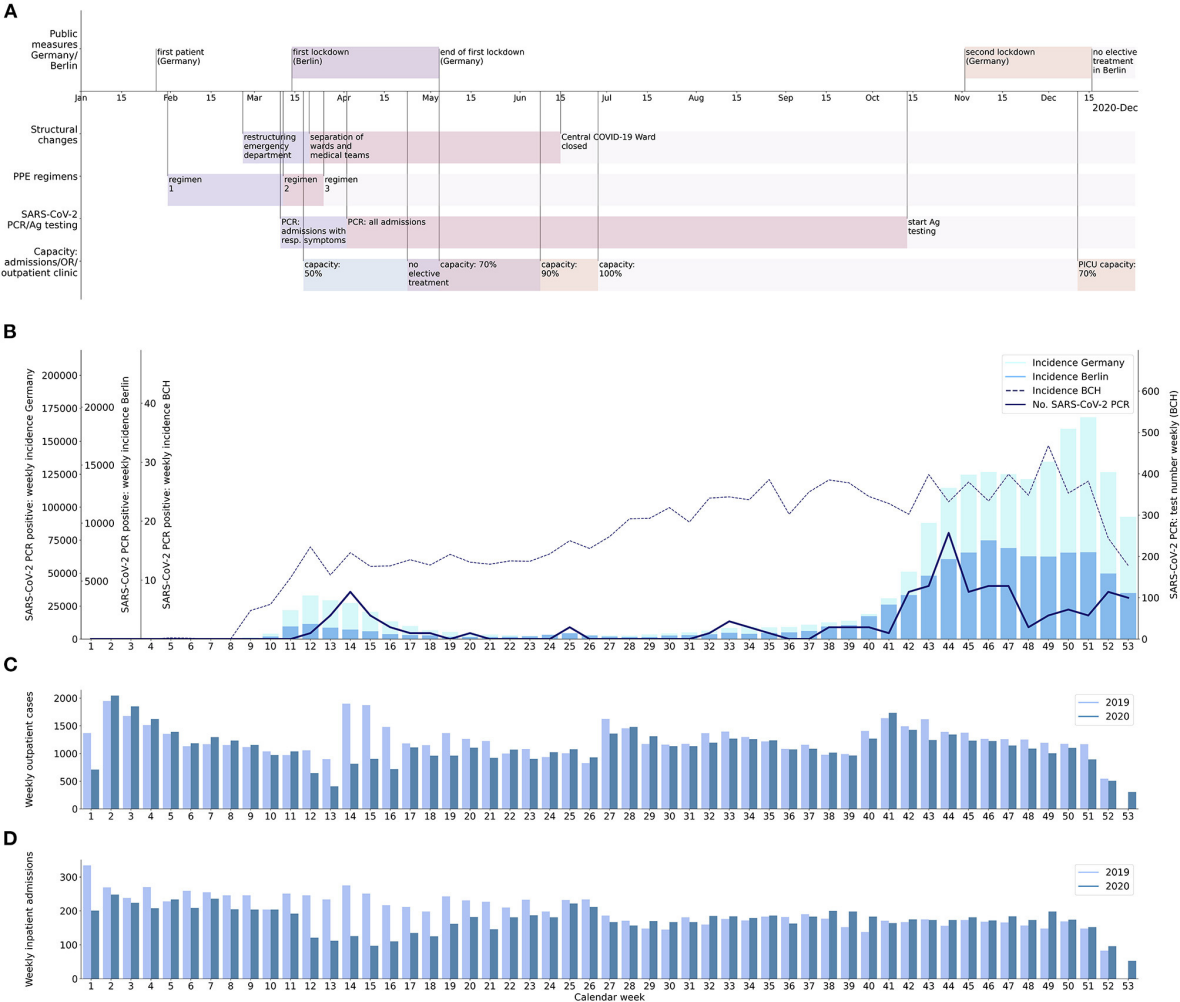
Procedural changes at BCH in relation to changes in incidence of SARS-CoV-2 infection and inpatient and outpatient case numbers. **(A)** Timeline with an overview on internal and external measures imposed as a response to the COVID-19 pandemic; first row: events and public measures in Germany or Berlin; second row: reorganization of wards and staff; third row: personal protective equipment (PPE) regimens (for description of regimens, see Table 1); fourth row: SARS-CoV-2 PCR and SARS-CoV-2 Ag testing strategies; fifth row: limitation of inpatient treatment, outpatient treatment and operation room (OR) capacities; PICU: pediatric intensive care unit. **(B)** Incidence of SARS-CoV-2 infection per calendar week in Germany (bright bars), Berlin (dark bars), and at BCH (thick line), and number of SARS-CoV-2 PCR tests undertaken on inpatients or patients to be admitted per calendar week (dashed line). **(C)** Number of outpatient cases at BCH per calendar week in 2019 (bright bars) and 2020 (dark bars). **(D)** Number of inpatient admissions at BCH per calendar week in 2019 (bright bars) and 2020 (dark bars); there were only 52 calendar weeks in 2019, whereas 53 calendar weeks in 2020.

According to the weekly incidence of SARS-CoV-2 infections we defined two waves of the COVID-19 pandemic in Berlin, Germany (referred to as “first wave” and “second wave”; Figure 1). The first wave covered the time between the first confirmed local case (calendar week 9, 2020) and the first week with a minimal incidence of SARS-CoV-2 infections that was followed by two consecutive weeks with increasing incidence of SARS-CoV-2 infections in Berlin (calendar week 20, 2020). The start of the second wave was defined as the last week with a minimal incidence of SARS-CoV-2 infections that was preceded by two consecutive weeks with decreasing incidence in Berlin (calendar week 28, 2020). The end of the second wave was arbitrarily defined as the week of the first vaccination against SARS-CoV-2 in Germany (calendar week 52, 2020).

### Governance and Communication

*First wave*: In February, 2020, after the first case of infection with SARS-CoV-2 in Germany, incident command teams were installed (i) at the institutional level [Charité—Universitätsmedizin Berlin (Charité)], (ii) at the hospital level (BCH) and (iii) across all Intensive Care Units at Charité (including PICU). During the first wave, conference calls of each of the incident command teams took place daily and procedural changes agreed on were communicated via e-mail, personal communication with the ward managers, and central procedural instructions published on the intranet of Charité.

*Changes during second wave*: During the second wave, conference calls of the same incident command teams took place at a lower frequency (once to three times weekly).

### Triage and Isolation Strategy

*First wave:* As in other hospitals around the world, hospital structure and patient flows at BCH were reorganized (9–19). The Pediatric Emergency Department was restructured. We separated the entrance to the Emergency Department from the main entrance of the BCH building and established one way patient routes. At the beginning of the first wave, emergency medical staff and rooms within the Emergency Department were divided into separate units. The first unit cared for patients with any signs of an infection or proven infection with SARS-CoV-2. The second one cared for immunocompetent patients without signs of an infection, the third unit for immunocompromised patients. This structure was maintained throughout the pandemic. The general pediatric IMC unit is located next to the Pediatric Emergency Department and has five isolation rooms. It was converted into a pediatric SARS-CoV-2/COVID-19-pending confirmation ward (referred to as “Central COVID-19 Ward”) during the first wave. All inpatients with unclear SARS-CoV-2 status and without a need for intermediate or intensive care were admitted to the Central COVID-19 Ward and only transferred to other pediatric wards upon negative result of SARS-CoV-2 PCR (“centralized admission”). All admitted and SARS-CoV-2 PCR positively tested patients stayed on the Central COVID-19 Ward. Previously hospitalized patients with a first positive SARS-CoV-2 PCR were transferred to the Central COVID-19 Ward. In the case of lacking capacity on the Central COVID-19 Ward, patients with pending SARS-CoV-2 results were admitted to one of two isolation rooms on the pediatric infectious diseases ward. Immunocompromized patients undergoing hemato-oncological treatment and awaiting admission remained in the Pediatric Emergency Department until SARS-CoV-2 PCR results became available. Upon a negative SARS-CoV-2 PCR result they were transferred directly to the pediatric Hemato-Oncology Unit, bypassing the Central COVID-19 Ward. The PICU was divided into two corridors, one COVID-19 corridor with two isolation rooms, and the rest of the PICU. Patients with pending SARS-CoV-2 PCR result or SARS-CoV-2 PCR positive patients with an indication for intermediate or intensive care were admitted or transferred to the COVID-19 corridor of the PICU. The maximum capacity of patients per room was reduced to two patients on all wards except the Central COVID-19 Ward (one patient).

*Changes during second wave*: The Central COVID-19 Ward/general pediatric IMC unit remained the ward on which patients with a confirmed SARS-CoV-2 infection were isolated. However, at the end of the first wave, the admission process was decentralized and remained in the decentralized mode throughout the second wave. The Central COVID-19 Ward was converted back to a general pediatric IMC unit. Admission of patients whose SARS-CoV-2 PCR results were pending was allowed to any other ward at BCH (“decentralized admission”).

### Testing for SARS-CoV-2

As soon as diagnostic testing by RT-PCR for SARS-CoV-2 (referred to as “SARS-CoV-2 PCR”) became available, all patients presenting to the Pediatric Emergency Department with respiratory symptoms were tested through a SARS-CoV-2 PCR of a naso- and oropharyngeal swab sample. When it became obvious that a large fraction of pediatric patients infected by SARS-CoV-2 was asymptomatic, the indication of SARS-CoV-2 PCR was widened to all patients planned for hospital admission (20, 21). A single laboratory (Labor Berlin GmbH) performed all SARS-CoV-2 PCR tests on swab samples from our hospital throughout the pandemic. At the beginning of the pandemic, when testing by SARS-CoV-2 PCR had just become available, we received several (three of a total of 9,553 SARS-CoV-2 PCR tests) weakly positive [cycle threshold (CT) > 30] SARS-CoV-2 PCR results of asymptomatic patients that proved SARS-CoV-2 negative in a second, confirmatory SARS-CoV-2 PCR (performed 36 h later). Consequently, asymptomatic patients with a weakly positive first SARS-CoV-2 PCR (CT > 30) underwent a second SARS-CoV-2 PCR test within 36 h. Asymptomatic patients with a weakly positive (CT > 30) first, but negative second SARS-CoV-2 PCR were considered SARS-CoV-2 negative. Any patients with a positive SARS-CoV-2 PCR (CT ≤ 30), symptomatic patients with a weakly positive SARS-CoV-2 PCR (CT > 30), and asymptomatic patients with at least two weakly positive SARS-CoV-2 PCR tests (CT > 30) were considered SARS-CoV-2 positive. As long as admission for elective treatment was allowed during the first wave, patients awaiting elective treatment were only admitted if SARS-CoV-2 PCR performed within 48 hours prior to admission was negative. The testing strategy by SARS-CoV-2 PCR was not changed during the second wave.

### Emergency Care Only vs. Maintenance of Elective Care

*First wave*: To keep hospital resources (especially anesthesiologic staff and intensive care beds) available for patients with COVID-19, during the first wave, Charité focused almost exclusively on highly urgent and emergency admissions including pediatric services at BCH. Measures included the discontinuation of admissions for elective treatments and a reduction of operation room capacities by 50%. Specialized outpatient clinics were only available for urgent visits. Instead, telemedical visits were enabled via video calls. After Berlin had crossed the peak incidence of SARS-CoV-2 infection during the first wave, capacities for elective inpatient treatments, outpatient clinics, and operation room capacities were increased in a stepwise fashion.

*Changes during second wave:* Based on experience from the first wave, unlike the departments at Charité treating adults, BCH did not reduce the inpatient capacity of non-emergency treatments at the beginning of the second wave. To maintain tertiary and quaternary pediatric care, the reduction of non-emergency treatment capacities was delayed until staff of the PICU had be relocated to the adult COVID intensive care units to compensate for staff shortage there. According to a decision of the Federal State of Berlin, non-emergency treatments were interrupted in calendar week 51.

### Personal Protective Equipment (PPE) and Hygiene

A summary of PPE regimens is provided in Table 1 and regimen changes are listed in chronological order in Supplementary Table 1. During the first wave, PPE regimens were tightened stepwise, and subsequently remained constant throughout the pandemic. Training in using PPE was conducted in all departments. In contrast to measures introduced for adult patients at Charité that allowed only one visitor, patients below the age of 18 years were allowed to receive one visitor each day in addition to permanent rooming in of one parent/legal guardian. This regime was kept up throughout the pandemic.

**Table 1:** Regimens of personal protective equipment (PPE); see Supplementary Table 1 for chronology of employment of PPE regimens.

	**Regimen 1**	**Regimen 2**	**Regimen 3**
Permanently	-	-	Surgical mask
Mask during bedside care (patient suspected of SARS-CoV-2)	FFP2 mask	Surgical mask	Surgical mask
Mask during bedside care with enhanced formation of aerosol (suctioning, endotracheal intubation, bronchoscopy, cardiopulmonary reanimation; patient suspected of SARS-CoV-2)	FFP3 mask with exhalation valve	FFP2 mask	FFP2 mask
Other PPE	Impermeable PPE gown, rubber gloves, safety glasses or goggles	Impermeable PPE gown, rubber gloves, safety glasses or goggles	Impermeable PPE gown, rubber gloves, safety glasses or goggles

### Reorganization of Hospital Staff

Due to closed specialized outpatient clinics and reduced operation room capacity during the first wave, medical and nursing staff were released to be relocated to the Central COVID-19 Ward and the PICU, to get familiar with these units and to allow reduction of overtime of these units' permanent staff. At the beginning of the first wave, medical teams of late and night shifts in the Pediatric Emergency Department and on the pediatric wards were divided into one team that cared for patients with positive or pending SARS-CoV-2 PCR result, and a second team that cared for all other patients. This regime was maintained throughout the pandemic. Further details on procedural changes are provided in Supplementary Table 1.

### Statistical Analyses

As indicators for the use of hospital resources within BCH during the COVID-19 pandemic, we used the inpatient and outpatient case numbers of BCH per calendar week. The according weekly numbers of the equivalent calendar weeks of the previous year (2019), prior to the pandemic, were used as a reference. As an indicator for the success of hygiene and isolation measures of BCH during the COVID-19 pandemic, we used two parameters: First, the overall number of nosocomial infections of patients by SARS-CoV-2 at BCH. Second, the monthly ratio of days of unscheduled sick leave or quarantine per full-time position (referred to as “sick rate”) of the medical and nursing staff of the departments at BCH that treated pediatric inpatients infected with SARS-CoV-2 (Pediatric Emergency Department, general pediatric IMC ward alias Central COVID-19 Ward, and PICU). The monthly sick rate of the equivalent months of 2019 was used as a reference. We used Shapiro-Wilk test to test for normal distribution. Both patient case numbers and sick rates followed normal distribution. We then used Levene test to test for homoscedasticity. All data sets compared showed equal population variance. Consequently, we used the two-tailed unpaired two sample *t*-test to test for statistical significance of the observed results. All data concerning case numbers, SARS-CoV-2 PCR testing, results of SARS-CoV-2 testing of inpatients, and sick rates was derived from the hospital's documentation system (SAP SE, Walldorf, Germany; POLYPOINT AG, Guemligen, Switzerland). All data was analyzed and visualized using Python v3.7 and its libraries (open source).

## Results

### Patient Admissions at BCH During the COVID-19 Pandemic

The mean number of admissions per week during the first wave was 147.5 and, thus, 37% lower compared to 234.0 admissions during the equivalent weeks in 2019 (*p* < 0.001; Figure 1). The minimum weekly number of admissions during the first wave was 97 (61% lower) in calendar week 15, compared to 251 in calendar week 15 of 2019. The mean weekly number of outpatient cases during the first wave was 899.3 (29% lower) compared to 1,274.0 in 2019 (*p* = 0.003). The minimum weekly number of outpatient cases during the first wave was 407 (55% lower) in calendar week 13, compared to 899 in calendar week 13 of 2019. The mean weekly number of inpatient admissions during the second wave (173.5) was 18% higher than during the first wave (*p* = 0.01) and was even slightly higher (7.0%) than the mean weekly number of admissions during the equivalent calendar weeks of 2019 (162.2; *p* = 0.06). The mean weekly number of outpatient cases during the second wave (1,174.3) was 31% higher than during the first wave (*p* = 0.001), but slightly lower (5.9%) than the mean weekly number of outpatient cases during the equivalent weeks of 2019 (1,248.4; *p* = 0.25).

### Number of Admitted Patients With SARS-CoV-2 Infection and Nosocomial SARS-CoV-2 Infections

Within the observation period, 37 inpatients infected with SARS-CoV-2 were treated at BCH (0.49% of inpatient cases). Nine of them were admitted during the first wave (0.47% of inpatient cases in this period). Twenty-eight of them were admitted during the second wave (0.63% of inpatient cases in this period; see Figure 1). SARS-CoV-2 infection was the principal diagnosis in 12 of the patients. Seven of these patients suffered from chronic diseases that raise the likelihood of admission due to respiratory or infectious diseases. Clinical symptoms of SARS-CoV-2 infection were generally mild. Seventeen Patients showed symptoms attributable to the infection with SARS-CoV-2. Six patients required supplementation of oxygen, of whom two required supplementary oxygen only during post-anesthesia care. Two adolescent patients admitted during the second wave required non-invasive ventilation, one of them obese, the other patient without a known risk factor. There was no nosocomial infection with SARS-CoV-2.

### Staff Availability in COVID Units and SARS-CoV-2 Infection of Staff

**Figure 2:**
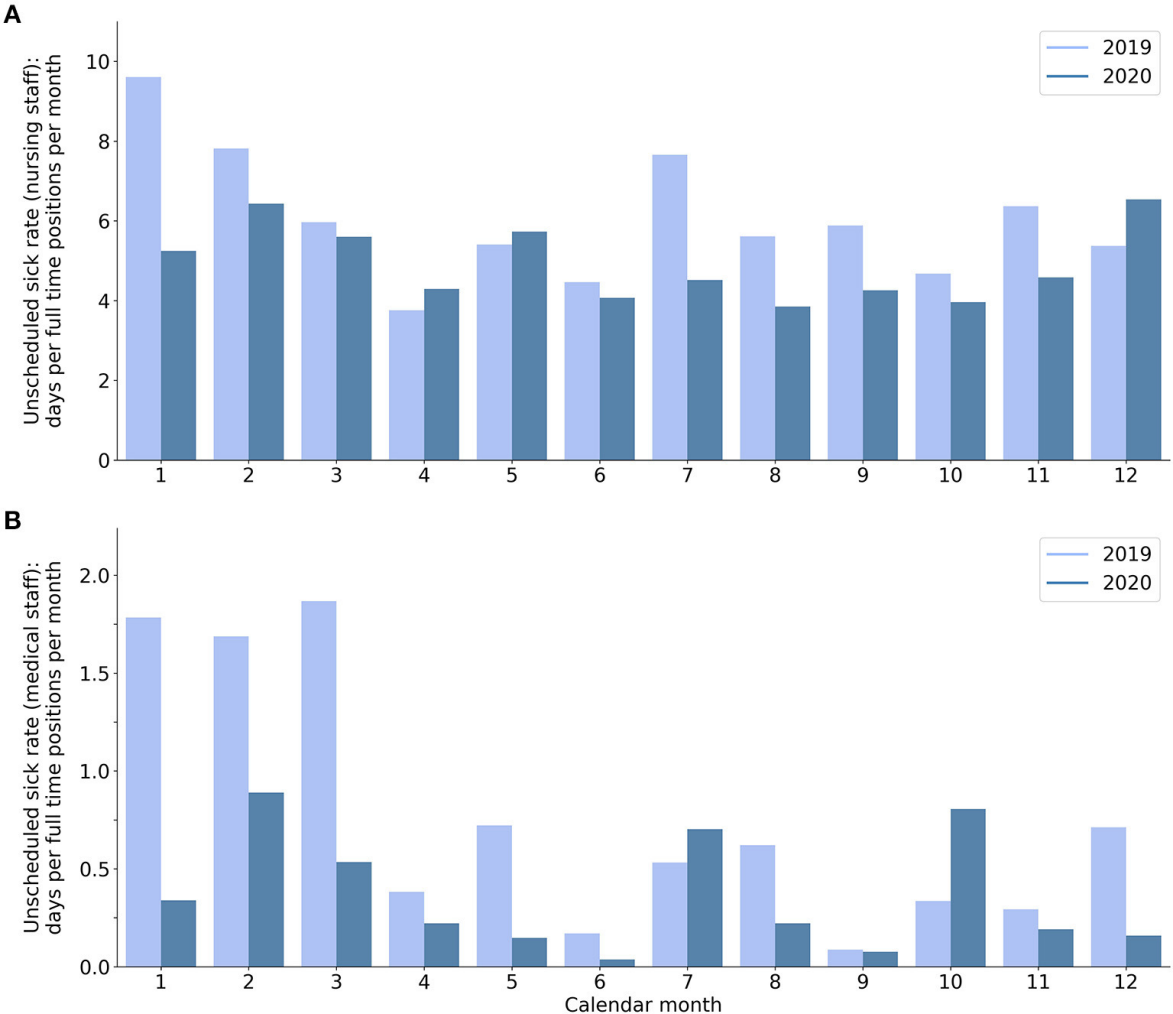
Unscheduled sick rate of **(A)** nursing staff and **(B)** medical staff at BCH departments caring for SARS-CoV-2 positive patients: days per full-time position per calendar month in 2019 (bright bars) and 2020 (dark bars).

Comparing the sick rate of the medical staff in the BCH departments caring for SARS-CoV-2 positive pediatric inpatients, there was a considerable decline of 53% (*p* < 0.001) in mean monthly days per full-time position from 2019 (0.77) to 2020 (0.36; Figure 2). A major part of this decline might be attributed to a high sick rate of medical staff during the first quarter of 2019, which occurred prior to and independent from the COVID-19 pandemic. Nevertheless, after excluding this first quarter of 2019 there is still a 33.5% (*p* < 0.001) decrease in the sick rate from 2019 (0.43) to 2020 (0.28). When comparing the sick rate of the nursing staff, there is a decline of 19% (*p* = 0.05) in mean monthly days per full-time position from 2019 (6.05) to 2020 (4.92; Figure 2). After excluding the first quarter in accordance with the medical staff there is still a 15% decrease (*p* = 0.11) from 2019 (5.47) to 2020 (4.65). During the first two waves of the pandemic, two nurses and one physician were infected by SARS-CoV-2. All of these infections with SARS-CoV-2 were clearly attributable to either external contacts (family members) or traveling abroad. There was no SARS-CoV-2 infection of staff attributable to in-hospital contacts, neither during the first nor during the second wave.

### Number of SARS-CoV-2 PCR Tests

The weekly number of SARS-CoV-2 PCR tests performed in patients prior to admission and inpatients substantially increased during the first weeks of the pandemic through calendar week 12. After this time, it constantly stayed above 150 tests per week and further increased to a maximum of 468 tests per week during the second wave. There was no decline of the weekly number of SARS-CoV-2 PCR tests between the first and second wave. The rate of positive SARS-CoV-2 PCR tests was 1.57% during the first and 1.62% during the second wave.

## Discussion

This study compares different approaches in response to the COVID-19 pandemic undertaken in the largest University Children's Hospital in Germany. These approaches comprise a centralized admission process and interruption of elective treatment during the first wave, and a decentralized admission process and maintenance of non-emergency treatments during the second wave. Specifically, we compared the numbers of admitted patients, in-hospital nosocomial SARS-CoV-2 infection and staff sick rates for these two different approaches using data from the year prior to the pandemic as reference. Inpatient and outpatient case numbers decreased during the period of a centralized admission process and limited elective treatment of non-COVID-19 patients in the first wave. In contrast, inpatient and outpatient case numbers showed less year by year deviation during the second wave due to maintenance of a decentralized admission process and continued provision of specialized non-emergency treatment to non-COVID-19 patients, without risk of nosocomial spread of SARS-CoV-2 infection among patients or staff. Our data support that maintenance of elective care is feasible during the COVID-19 pandemic with the SARS-CoV-2 testing strategy and hygiene measures implemented at our hospital.

Numerous pediatric hospitals in Europe, Asia and America discontinued elective inpatient and outpatient treatments similar to our approach during the first wave of the COVID-19 pandemic (Supplementary Tables 2, 3) (9–19). In a survey including 102 pediatric hospitals in Europe, >90% had canceled elective treatments (18). The majority of reports states that elective specialized pediatric surgeries were canceled. Further, several reports describe the establishment of a centralized admission process involving a dedicated unit or ward for admitted pediatric patients with a pending SARS-CoV-2 PCR test result (9–13, 15, 19). However, the consequences of maintaining elective pediatric care and a decentralized admission process during a period with high incidence of SARS-CoV-2 infection in the local population (maximum 7 day incidence in Berlin > 220/100,000) on the numbers of patients that can be treated and the risk of nosocomial infections have not been evaluated systematically. In comparison to patient case numbers in the same calendar weeks of the previous year, the numbers of both inpatient admissions and outpatient cases at BCH declined substantially during the first wave of the pandemic (calendar weeks 9–20). This decline was probably related to a combination of internal and external factors. Internal factors include the early discontinuation of non-emergency inpatient and outpatient treatments. External factors include public measures such as restrictions in social life leading to less infections and trauma, but also fear of SARS-CoV-2 infection in hospitals that made parents avoid medical consultations (22–24). During the second wave (calendar weeks 28–52), weekly numbers of inpatient admissions and outpatient cases were higher compared to the first wave. Weekly numbers of inpatient admissions during the second wave were even slightly higher than in the corresponding weeks of the previous year. This observation may be explained by several factors including maintenance of BCH's non-emergency tertiary and quaternary care, backlog of disease burden of patients that could not be treated during the first wave, and less fear of SARS-CoV-2 infection in the hospital and thus less avoidance of outpatient appointments or hospital admissions by parents. The decision to continue provision of specialized care for elective treatment of non-COVID-19 patients during the second wave was based on the following considerations. First, during the first wave, it became evident that the released hospital capacity is not required for the care of pediatric COVID-19 patients due to the generally mild clinical presentation in children and adolescents and that nosocomial infections can be effectively controlled by the hygiene measures that were put in place. Second, there was a growing concern among parents and physicians that the lack of regular outpatient follow-up and necessary inpatient treatment may aggravate chronic diseases (25, 26). Telemedicine, which had been installed in most outpatient clinics, could not replace face to face appointments. Third, there was an urgent need to perform surgical treatments that had to be deferred during the first wave (17). Finally, a second period of unutilized resources as during the first wave would have led to a substantial financial deficit caused by fixed expenses.

Despite continuation of elective treatment of non-COVID-19 patients and a year by year increase in inpatient admissions during the second wave, we observed no nosocomial infections with SARS-CoV-2. Further, the sick rate of staff of departments caring for SARS-CoV-2 positive patients did not increase during the COVID-19 pandemic compared to the previous year. These observations may in part be explained by the low fraction of SARS-CoV-2 positive patients of all inpatients treated during the pandemic (0.47% during the first and 0.63% during the second wave). However, our observations show that basic internal hygiene measures that were established initially and maintained throughout the pandemic (including personal protective equipment, isolation rooms, and extensive SARS-CoV-2 PCR testing) were effective to prevent cross-infection among patients and staff. With these measures in place, despite a rather high incidence above 200/100,000 in the general population BCH did not require a centralized admission process via the Central COVID-19 Ward to avoid in-hospital infections with SARS-CoV-2.

### Limitations

Structure and the availability of services differ between pediatric hospitals, making it difficult to generalize observations made at BCH. Furthermore, due to the surge of SARS-CoV-2 virus variants such as delta with increased risk of transmission, measures presented here might prevent nosocomial SARS-CoV-2 infections less effectively during future waves than during the first two waves of the pandemic. Future waves of the pandemic might also coincide with increased circulation of other viruses than SARS-CoV-2, which might lead to higher amounts of patients with respiratory symptoms (27). Therefore, limited hospital capacity might interfere with the triage and isolation measures presented here.

### Conclusion

During the first wave of the COVID-19 pandemic, numerous pediatric hospitals canceled elective inpatient and outpatient treatments and established a dedicated ward for admitted patients with pending SARS-CoV-2 PCR test results. To our knowledge, this is the first study that evaluates these internal measures of a pediatric hospital in response to the pandemic. Our data of a large tertiary and quaternary pediatric hospital supports that maintaining elective specialized care may be safe and optimize the use of hospital resources during the COVID-19 pandemic. Further, our data indicates that a centralized admission process via a dedicated ward for admitted patients with pending SARS-CoV-2 PCR test results may be dispensable for the prevention of in-hospital SARS-CoV-2 infection in pediatric hospitals. Given the successful prevention of in-hospital SARS-CoV-2 infection and low incidence of severe COVID-19 that requires hospital admission in children and adolescents, pediatric hospitals of tertiary and quaternary care may remain focused on specialized care rather than discontinue their services for non-COVID-19 patients to release hospital capacity for the treatment of SARS-CoV-2-positive pediatric patients. This approach may help to mitigate unwanted sequelae related to temporary suspension and withholding of specialized care to child and adolescent health.

## Data Availability Statement

The datasets presented in this article are not readily available because they consist of identifiable data. Requests to access the datasets should be directed to Alexander Gratopp, alexander.gratopp@charite.de.

## Ethics Statement

Ethical review and approval was not required for the study on human participants in accordance with the local legislation and institutional requirements. Written informed consent from the participants' legal guardian/next of kin was not required to participate in this study in accordance with the national legislation and the institutional requirements.

## Author Contributions

NT collected data, designed instruments for the dat a analyses, carried out the data analyses, drafted the initial manuscript, reviewed, and revised the manuscript. NT, HB, and AG conceptualized the study. AR provided data and carried out data analyses. AR, AK, UR, KL, MM, HB, and AG managed the hospital's response to the COVID-19 pandemic. AK, UR, KL, MM, HB, and AG reviewed and revised the manuscript. All authors contributed to the article and approved the submitted version.

## Conflict of Interest

HB was employed by Labor Berlin GmbH, a joint subsidiary of the public hospitals Charité Universitätsmedizin Berlin and Vivantes—Netzwerk für Gesundheit GmbH. The remaining authors declare that the research was conducted in the absence of any commercial or financial relationships that could be construed as a potential conflict of interest.

## Publisher's Note

All claims expressed in this article are solely those of the authors and do not necessarily represent those of their affiliated organizations, or those of the publisher, the editors and the reviewers. Any product that may be evaluated in this article, or claim that may be made by its manufacturer, is not guaranteed or endorsed by the publisher.
