# Supplementary material for: Maintenance of Elective Patient Care at Berlin University Children's Hospital During the COVID-19 Pandemic
Source: Front Pediatr. 2021 Aug 30;9:694963. doi: 10.3389/fped.2021.694963 (PMC8435743; doi:10.3389/fped.2021.694963)
Supplement: Supplementary file 1 [file Data_Sheet_1.pdf]

Maintenance of elective patient care at Berlin University Children’s Hospital during the COVID-19 pandemic

Supplementary tables

Supplementary table 1

Date	Germany/Berlin	Berlin University Children’s Hospital (BCH)				
		Governing and communication	Triage and isolation	Hygiene/ PPE regimens	SARS-CoV-2 PCR	Admission/ OR/ outpatient clinic capacity
27 Jan	first SARS-CoV-2 PCR positive case in Germany					
31 Jan			risk patients (respiratory symptoms and one of two: history of stay in region at risk or contact to SARS-CoV-2 PCR positive person) in emergency unit: treatment in isolation room	staff: regimen 1 when treating patients with risk factors for COVID-19; patients with risk factors: surgical mask; parents/ legal guardians: surgical mask		
26 Feb		launch of incident command teams (levels: Charité, Charité-intensive care units, BCH)	separate entrance to pediatric emergency unit for patients suspected of COVID-19			
27 Feb					inclusion of SARS-CoV-2 PCR whenever swab testing of patients with respiratory symptoms	
03 Mar	prohibition of export of personal protective equipment					
10 Mar	prohibition of events with > 1000 participants				SARS-CoV-2 PCR on all pediatric patients with respiratory symptoms or admission to PICU	
11 Mar				staff: regimen 2 during close contact (distance < 2 m) to patients with risk factors		

12 Mar		extension of SARS-CoV-2 PCR capacity	separate entrance to pediatric emergency unit for all emergency patients			discontinuation of elective admission of pediatric patients with respiratory symptoms
13 Mar				limitation of visits: one parent/ legal guardian permanently + one visitor per day per inpatient		
14 Mar	beginning of local lockdown in Berlin					
16 Mar						reduction of OR capacity to 75 %, limitation of elective surgeries
18 Mar						reduction of OR capacity to 50 %
20 Mar		separation of medical late and night shifts (infectious team/ non-infectious team)	launch of centralized admission process: Central COVID-19 ward and COVID-19 corridor of PICU			
		first SARS-CoV-2 PCR positive patient at BCH	pediatric emergency unit: separate area for treatment of hemato-/oncology patients			
22 Mar	beginning of countrywide lockdown in Germany					
25 Mar				staff: regimen 3		
30 Mar		SARS-CoV-2 PCR result within 24 hours				
02 Apr					SARS-CoV-2 PCR on all admitted patients and all co-admitted parents/ legal guardians	
15 Apr			extension of Central COVID-19 Ward			

22 Apr						requirement for elective admissions: SARS-CoV-2 PCR negative (test max. 48 hours prior to admission)
23 Apr						no elective treatments till 04 May
27 Apr				visitors: surgical mask		
04 May	end of local and countrywide lockdown in Berlin and Germany	launch of SARS-CoV-2 serology testing				restart of elective admissions and surgery<br>increase of capacities of OR and outpatient clinics to 50 %
30 May						increase of capacities of OR and outpatient clinics to 75 %
08 Jun						increase of capacities of OR and outpatient clinics to 90 %
15 Jun			reconversion of Central COVID ward to general pediatric IMC ward			
12 Aug		first SARS-CoV-2 PCR positive patient of second wave in pediatric departments				
13 Aug			designated pavilion outside BCH building for nasopharyngeal swabs on patients scheduled for elective admission			
13 Oct		first SARS-CoV-2 Ag test in pediatric emergency unit				
02 Nov	second lockdown (GER): light restrictions	beginning of redistribution of PICU nursing staff to adult COVID intensive care units				closure of one pediatric surgery ward

11 Dec				limitation of visits (one parent/ legal guardian or one visitor per day), exceptions for inpatients > 7 d hospital stay or prefinal children		reduction of PICU capacity to 70 %
16 Dec	second lockdown (GER): severe restrictions					
17 Dec	cessation of non-emergency treatments in all hospitals in Berlin					
27 Dec	start of vaccination against SARS-CoV-2 in Berlin					

Supplementary table 1. Chronology of measures; columns left to right: events and public measures (Germany countrywide, Berlin statewide); governing and communication; structural changes due to triage and isolation stretagies; hygiene and personal protective equipment regimens; indication of SARS-CoV-2 PCR on nasopharyngeal swab; limitation of OR capacity, outpatient visits, and inpatient admissions.

Supplementary table 2

Publication	Location/city, country	Pediatric hospital: number of patient cases	Pediatric hospital: number of inpatient beds	Referral region: number of inhabitants	Tertiary care	Quaternary care
Buonsenso et al., 2020 (11)	Rome, Italy	~1000 outpatient visits per week	unknown	unknown	yes	unknown
Bressan et al., 2020 (18)	Europe (multinational, 102 hospitals)	not applicable	not applicable	not applicable	unknown	unknown
Dona et al., 2020 (9)	Padua, Italy	26000 pediatric emergency department visits per year	unknown	0.35 million	unknown	unknown
Giamouris et al., 2020 (12)	Athens, Greece	unknown	118	unknown	yes	unknown
Meier et al., 2020 (15)	Cincinnati, USA	unknown	unknown	unknown	unknown	unknown
Nicastro et al., 2020 (14)	Bergamo, Italy	unknown	36 given (17 general ped, 8 hemato-oncol., 11 solid organ transplant programm)	1.1 million	yes	unknown
Parikh et al., 2020 (16)	Seattle, USA	unknown	407 beds	11 million	yes	yes
Skarsgard et al., 2020 (17)	Canada (countrywide)	not applicable	not applicable	not applicable	yes	yes
Thampi et al., 2020 (10)	Singapore, Singapore	unknown	1200 beds (whole hospital, only partly pediatric beds), 18 beds (PICU)	unknown	yes	unknown
Zeng et al., 2020 (19)	Sichuan province, China	unknown	40 beds (PICU), 1657 beds (women‘s and children‘s hospital)	unknown	yes	unknown
Zhang et al., 2020 (13)	Shanghai, China	unknown	unknown	unknown	unknown	unknown

Supplementary table 2. Synopsis of publications regarding measures in pediatric hospitals as a response to the COVID-19 pandemic: baseline data of hospitals.

Supplementary table 3

Publication	Central admission via one ward or unit	Discontinuation of non-emergency treatment	Do measures affect all pediatric departments?	Number of SARS-CoV-2 PCR positive patients	Number of nosocomial infections by SARS-CoV-2 (staff)	Number of nosocomial infections by SARS-CoV-2 (patients)	Comparison of measures regarding outcome
Buonsenso et al., 2020 (11)	yes (clinical signs suggestive of COVID-19: admission to dedicated pediatric COVID-19 ward)	yes (outpatients: cancelation of all non-urgent visits; inpatients: unknown)	yes	3 % of 90 clinically suspected	unknown	unknown	no
Bressan et al., 2020 (18)	unknown	yes (>90% of hospitals: cancelation of planned treatments)	not applicable	unknown	unknown	unknown	no
Dona et al., 2020 (9)	yes (suspicion of COVID-19: central admission to isolation area; no suspicion: decentral admission)	unknown	no	16 of 1184 patients	unknown	unknown	no
Giamouris et al., 2020 (12)	yes (suspected cases (fever/resp. symptoms): central admission; non-suspected cases: unknown)	unknown	yes	1 of 120 suspected patients	unknown	unknown	no
Meier et al., 2020 (15)	yes (special unit for patients with COVID-19 or under investigation)	yes (elective treatments canceled)	yes	unknown	unknown	unknown	no
Nicastro et al., 2020 (14)	no	unknown	yes (emphasis on emergency department)	17 (of 58 naso-/ oropharyngeal swabs)	6 confirmed, 16 suspected cases before adoption of protocol; 4 cases after adoption of protocol	unknown	no

Parikh et al., 2020 (16)	no	yes (all non-urgent cases canceled, ambulatory center closed)	yes	unknown	unknown	unknown	no
Skarsgard et al., 2020 (17)	unknown	yes (scheduled surgery: restricted to urgent procedures)	not applicable	unknown	unknown	unknown	no
Thampi et al., 2020 (10)	yes (suspected cases (resp. tract infection): admission to negative pressure room on PICU (2 negative swabs (second min. 24h after first) to leave negative ppressure room))	yes (all elective surgeries canceled)	yes (emphasis on PICU)	unknown	unknown	unknown	no
Zeng et al., 2020 (19)	yes (high suspicion of COVID-19: transfer to negative pressure ward)	unknown	no, only PICU	unknown	unknown	unknown	no
Zhang et al., 2020 (13)	yes (transition room for people from outside Shanghai; COVID ward: dedicated quarantine ward and divided into three zones (clean, semi-dirty, dirty))	unknown	yes	61 confirmed, 92 suspected	0	0	no

Supplementary table 3. Synopsis of publications regarding measures in pediatric hospitals as a response to the COVID-19 pandemic: structural and procedural changes during COVID-19 pandemic, data on infection of patients/staff with SARS-CoV-2, and whether outcome of measures in response to pandemic is reported.
